# Fabricating Spinel-Type High-Entropy Oxides of (Co, Fe, Mn, Ni, Cr)_3_O_4_ for Efficient Oxygen Evolution Reaction

**DOI:** 10.3390/ma17143415

**Published:** 2024-07-10

**Authors:** Xiaofei Hao, Ran Wang, Xiumin Tan, Xiufeng Zhang, Xupo Liu, Zhaoyang Wu, Dongli Yuan

**Affiliations:** 1 Zhengzhou Institute of Multipurpose Utilization of Mineral Resources, Chinese Academy of Geological Sciences, Zhengzhou 450006, China; zzs_tan@163.com (X.T.); zh200318@126.com (X.Z.); wzy500@sina.com (Z.W.); yuandongli99@163.com (D.Y.); 2 School of Materials Science and Engineering, Henan Normal University, Xinxiang 453007, China; 2125283034@stu.htu.edu.cn

**Keywords:** spinel type high-entropy oxides, electrocatalyst, oxygen evolution reaction, water splitting

## Abstract

Fabricating efficient oxygen evolution reaction (OER) electrocatalysts is crucial for water electrocatalysis. Herein, the spinel-type high-entropy oxides of (Co, Fe, Mn, Ni, Cr)_3_O_4_ were synthesized through the high-temperature calcination approach. The influences of calcination temperatures on structures and electrochemical properties were investigated. The optimized catalyst of HEO-900 contains the hybrid structure of regular polyhedrons and irregular nanoparticles, which is beneficial for the exposure of electrochemically active sites. It was identified that the abundant high-valence metal species of Ni^3+^, Co^3+^, Fe^3+^, Mn^4+^, and Cr^3+^ are formed during the OER process, which is generally regarded as the electrochemically active sites for OER. Because of the synergistic effect of multi-metal active sites, the optimized HEO-900 catalyst indicates excellent OER activity, which needs the overpotential of 366 mV to reach the current density of 10 mA cm^−2^. Moreover, HEO-900 reveals the prominent durability of running for 24 h at the current density of 10 mA cm^−2^ without clear delay. Therefore, this work supplies a promising route for preparing high-performance multi-metal OER electrocatalysts for water electrocatalysis application.

## 1. Introduction

Water electrolysis is a promising strategy for producing clean and renewable hydrogen energy fuel through employing sustainable solar and wind energy [1,2,3]. During the electrolysis process, the oxygen evolution reaction (OER) is a crucial step as it determines the overall efficiency and performance of the electrolyzer [4,5]. Thus, research on high-performance OER catalysts for water electrolysis is of great significance. Generally, efficient and cost-effective OER catalysts are essential to improve the kinetics of oxygen evolution, reduce energy consumption, and enhance the overall electrolyzer performance [6,7]. Recent advances have reported large numbers of electrocatalysts for OER applications [8,9]. However, taking both the prominent activity and durability of OER catalysts into consideration at the same time is still a huge challenge.

There are many compounds being investigated for OER, such as transition metal-based hydroxides [7], oxides [10], phosphides [11,12], sulfides [13,14,15] and nitrides [16]. Among them, spinel oxides (AB_2_O_4_) have been widely used as an electrocatalyst for oxygen evolution due to their diverse and adjustable electronic structure and properties [17]. The development of spinel-type catalysts for OER has gained significant attention in the field of electrocatalyst exploration [18]. Over the years, extensive efforts have been made to optimize the composition and structure of spinel catalysts. Different synthesis techniques, including sol-gel, hydrothermal, and solid-state methods, have been employed to fabricate catalysts with desirable properties [19]. The addition of various dopants or nanoscale modifications has also been explored to improve catalytic performance. However, the electrocatalytic properties of spinel-type catalysts are still inferior to precious metal-based catalysts (RuO_2_ and IrO_2_) [20]. Fortunately, the construction of high-entropy oxides is an efficient approach to enhance the electrochemical performances of spinel oxides. High-entropy oxides are a series of compounds with five or more different species with equal atomic ratios [21,22]. Taking the prominent advantage of the high-entropy synergetic effect of multiple metallic oxides is beneficial for tailoring the electronic structures and OER catalytic activity of spinel-type oxides [21]. In addition, spinel-type high-entropy oxides always possess high electrochemical durability due to the high-entropy stabilization effect [23,24]. However, the high-entropy alloy nanoparticles always contain four or more elements, and the problem of low-product purity easily occurs, hindering their application in the electrocatalytic system. 

In this work, the wet ball-milling and following high-temperature calcination were adopted to synthesize the spinel-type high-entropy oxides of (Co, Fe, Mn, Ni, Cr)_3_O_4_. The wet ball-milling treatment could effectively promote the full mixing of raw material powders, which is conducive to the formation of high-purity, high-entropy oxides in the calcination process. The optimized catalyst displays the special hybrid structure of regular polyhedrons and irregular nanoparticles, which promotes the exposure of electrochemically active sites. Owing to the synergistic effect of the multi-metal sites, the as-prepared HEO-900 catalyst indicates excellent OER activity of just needing the overpotential of 366 mV to reach the current density of 10 mA cm^−2^. In addition, the HEO-900 catalyst exhibits prominent durability when running for 24 h at the current density of 10 mA cm^−2^ with clear delay. Therefore, this work supplies a promising route for preparing high-performance multi-metal OER electrocatalysts for water electrocatalysis application.

## 2. Materials and Methods

### 2.1. Materials

The raw materials of Co_2_O_3_, Fe_2_O_3_, Mn_2_O_3_, Ni_2_O_3_, and Cr_2_O_3_ with AR purity were all purchased from Tianjin Kemiou Chemical Reagent Co., Ltd. (Tianjin, China). KOH (85%) was obtained from Shanghai Aladdin Bio-Chem Technology Co., Ltd. (Shanghai, China). Carbon cloth was obtained from the Kunshan Tengerhui Co., Ltd. (Kunshan, China). Deionized water was applied to dispense the electrolyte solution. 

### 2.2. Electrocatalyst Preparation

The spinel-type high-entropy oxides of (Co, Fe, Mn, Ni, Cr)_3_O_4_ were synthesized through the high-temperature calcination method. Firstly, 0.2 mol Co_2_O_3_, 0.2 mol Fe_2_O_3_, 0.2 mol Mn_2_O_3_, 0.2 mol Ni_2_O_3_, and 0.2 mol Cr_2_O_3_ were ground with alcohol as the grinding medium for 3 h. Then, the mixtures were heated up to the temperature of x °C for 4 h with a heating rate of 5 °C min^−1^ in an air atmosphere. After cooling down to room temperature, the obtained catalysts were named HEO-x, where x represented the heating temperature of 800, 900, 1000, and 1100 °C, respectively. For comparison, the raw materials of oxide mixtures were also applied as the HEO-O catalyst.

### 2.3. Physical Characterization

The X-ray diffraction (XRD) patterns of the prepared electrocatalysts were performed through a Dutch Empyrean in situ electrochemical X-ray diffractometer with CuKa as the radiation source. The morphologies of catalysts were achieved on the Regulus8220 field emission scanning electron microscope (FESEM) and the JEOL JEM-2100 transmission electron microscope (TEM) equipped with energy-dispersive X-ray spectrum (EDS). The surface elemental compositions and element valences of samples were exploited via the Thermo Scientific (Waltham, MA, USA) K-Alpha X-ray photoelectron spectroscopy (XPS). 

### 2.4. Electrochemical Determinations

In this work, all the electrochemical determinations were carried out in a 1.0 M KOH electrolyte via a three-electrode system based on the CHI660E electrochemical workstation (Chenhua). To achieve the working electrode, 10 mg of the prepared catalyst, modified Kaolin and graphene (5:2:3) was uniformly dispersed in a 2 mL 0.1% Nafion/isopropanol solution through ultrasonic treatment. Then, 100 μL of the obtained ink was dropped on the treated carbon paper and dried to obtain the working electrode. The graphite rod and Hg/HgO electrode were employed as the counter electrode and reference electrode, respectively. All the potentials were revised according to the reversible hydrogen electrode (RHE) through the equation E (RHE) = E (Hg/HgO) + 0.0591 × pH + 0.098 V. Linear sweep voltammetry (LSV) curves of catalysts were detected in the voltage range of 1.0~1.8 V at a scan rate of 5 mV s^−1^. To evaluate the durability, the chronopotentiometric curve of HEO-900 was determined at the current density of 10 mA cm^−2^. The double-layer capacitance was estimated by cyclic voltammetry in the range of 0.75~0.85 V with sweep rates of 10, 20, 40, 60, 80, and 100 V s^−1^. Electrochemical impedance spectroscopy (EIS) data were determined in the frequency range of 0.1 MHz~0.01 Hz.

## 3. Results

### 3.1. Structure Characterization

**Scheme 1:**
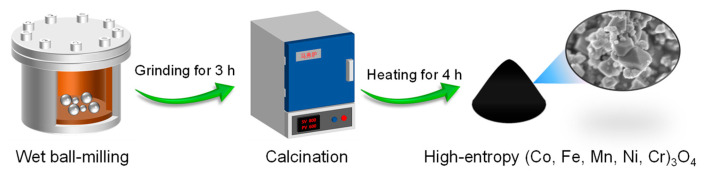
The synthesis process diagram of preparing the spinel-type high-entropy oxides of (Co, Fe, Mn, Ni, Cr)_3_O_4_.

**Figure 1:**
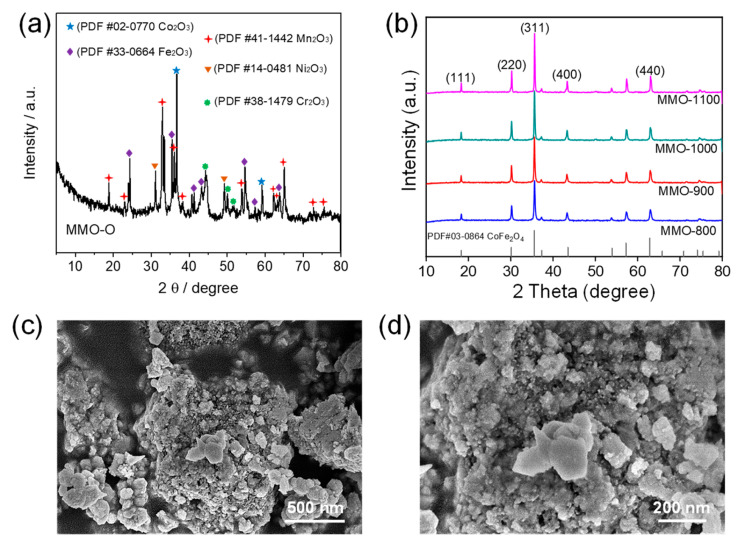
(**a**) XRD pattern of the raw material mixture. (**b**) XRD patterns of the prepared HEO-800, HEO-900, HEO-1000 and HEO-1100 catalysts. (**c**,**d**) SEM images of HEO-O.

The spinel-type high-entropy oxides of (Co, Fe, Mn, Ni, Cr)_3_O_4_ in this work were synthesized through the wet ball-milling and following high-temperature calcination, as shown in Scheme 1. The wet ball-milling treatment is believed to promote the sufficient mixing of raw material powders and thus accelerates the formation of high-entropy oxides in the calcination process. The obtained catalysts were named HEO-x, where x represents the heating temperature of 800, 900, 1000, and 1100 °C, respectively. Firstly, the phase compositions of catalysts were determined and analyzed. The raw material mixture of HEO-O displays the tanglesome XRD peaks, as shown in Figure 1a. The XRD peaks of Co_2_O_3_, Fe_2_O_3_, Mn_2_O_3_, Ni_2_O_3_, and Cr_2_O_3_ can be observed and consist of a composition of raw materials. After the calcination process, the XRD peaks changed significantly. All the samples of HEO-800, HEO-900, HEO-1000 and HEO-1100 showed similar XRD peaks located at 18.27°, 30.17°, 35.59°, 37.20°, 43.28°, 53.76°, 57.33°, 62.97° and 74.56°, as shown in Figure 1b. These peaks are identical to the standard characteristic peaks of CoFe_2_O_4_ (PDF 03-0864), revealing the single spinel phase. There is no peak belonging to the other oxides observed. Thus, the introduction of other metal elements (Ni, Mn, and Cr) clearly does not change the original crystal structure of the CoFe_2_O_4_ phase. According to the above result, we think that the as-synthesized high-entropy oxides of (Co, Fe, Mn, Ni, Cr)_3_O_4_ display a cubic phase crystal structure with the lattice parameter of a = 8.377 Å, similar to that of CoFe_2_O_4_ (PDF#03-0864). The XRD result preliminarily confirms the successful fabrication of high-entropy (Co, Fe, Mn, Ni, Cr)_3_O_4_ oxides with a single cubic spinel phase. In addition, it is notable that the XRD peak intensity of the catalysts shows an enhanced trend as the calcination temperatures increase, proving the promoted crystallinity of these high-entropy oxides.

**Figure 2:**
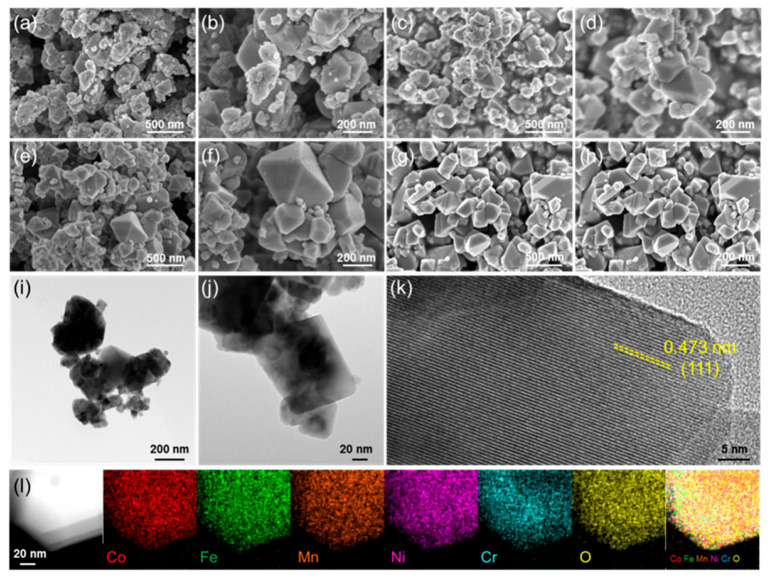
SEM images of (**a**,**b**) HEO-800, (**c**,**d**) HEO-900, (**e**,**f**) HEO-1000, (**g**,**h**) HEO-1100. TEM images of HEO-900: (**i**,**j**) TEM images, (**k**) high-resolution TEM images, and (**l**) elemental mapping images of Co, Fe, Mn, Ni, Cr, and O.

SEM images were detected to exploit the microstructure of the prepared electrocatalysts. The SEM images of HEO-O in Figure 1c,d indicate that the raw material mixture consists of some irregular grains with different sizes. However, the morphologies were clearly changed after the high-temperature calcination process, as shown in Figure 2a–h. It was found that there are some regular polyhedrons formed in these catalysts during the calcination process, and the percentages of polyhedrons are promoted as the calcination temperatures increase. The HEO-800 sample that is calcined at 800 °C still contains many irregular nanograins (Figure 2a,b). As the calcining temperature increases up to 900 °C, the number of irregular nanoparticles further decrease, and more polyhedrons are generated. The HEO-900 electrocatalyst shows the structure of regular polyhedrons with a small quantity of irregular nanoparticles (Figure 2c,d). When the temperatures further increase to 1000 °C and 1100 °C, the synthesized HEO-1000 and HEO-1100 samples mainly display the morphology of regular polyhedrons (Figure 2e–h). Thus, the calcination temperatures have an important effect on the microstructures of high-entropy (Co, Fe, Mn, Ni, Cr)_3_O_4_ electrocatalysts. In general, regular polyhedrons contain high degrees of crystallinity, which are damaging for promoting the exposure of electrochemically active sites. However, the hybrid structure of regular polyhedrons and irregular nanoparticles may be beneficial for achieving excellent OER activity.

The TEM images of the HEO-900 sample have also been determined to analyze the structure, as shown in Figure 2i–l. It can be seen that HEO-900 consists of some regular polyhedrons and irregular nanoparticles (Figure 2i–l), matching well with the SEM analysis. The lattice fringes with a lattice distance of 0.473 nm correspond to the (111) facet of the CoFe_2_O_4_ phase (Figure 2k). The mapping images indicate the uniform dispersion of Co, Fe, Mn, Ni, Cr, and O around the polyhedron (Figure 2l), further proving the successful fabrication of the spinel-type high-entropy (Co, Fe, Mn, Ni, Cr)_3_O_4_ electrocatalyst. The uniform dispersion of metal atoms is conducive to achieving the synergistic effect of multiple metal sites, which is expected to enhance the OER performances.

### 3.2. Electrocatalytic Performances

**Figure 3:**
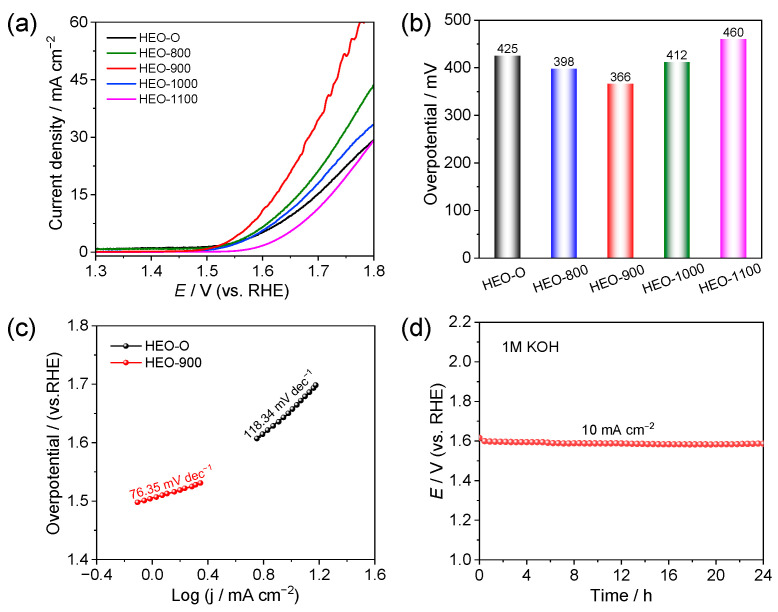
(**a**) OER polarization curves of the prepared catalysts. (**b**) Comparison of the overpotentials at the current density of 10 mA cm^−2^. (**c**) Tafel slopes of HEO-O and HEO-900. (**d**) The chronopotentiometric curve of HEO-900 at the constant current density of 10 mA cm^−2^.

The electrocatalytic performances of the high-entropy (Co, Fe, Mn, Ni, Cr)_3_O_4_ catalysts were determined in 1.0 M KOH through the common three-electrode system. Figure 3a shows the OER polarization curves of the prepared catalysts. It can be observed that the HEO-900 electrocatalyst displays the best OER activity among these samples. To achieve the current density of 10 mA cm^−2^, HEO-900 just needs the overpotential of 366 mV, which is much lower than those of HEO-O (425 mV), HEO-800 (398 mV), HEO-1000 (412 mV), and HEO-1100 (460 mV), as shown in Figure 3b. In addition, the Tafel slopes of HEO-O and HEO-900 calculated from the LSV curves were adopted to evaluate the OER activity (Figure 3c). It was found that the HEO-900 electrocatalyst possesses a lower Tafel slope of 76.35 mV dec^−1^ than HEO-O (118.34 mV dec^−1^), revealing a faster OER reaction mechanism and better catalytic activity. We compared the OER performances of HEO-900 with the recently reported catalysts, as shown in Table S1 [25,26,27,28,29,30,31,32,33,34,35,36]. It is notable that HEO-900 displayed compatible performances with the reported catalysts, revealing promising applications. The excellent OER activity of the high-entropy (Co, Fe, Mn, Ni, Cr)_3_O_4_ catalyst is due to the synergetic effect of multiple metal sites. In addition, durability is also important for the OER electrocatalysts. The chronopotentiometric curve of HEO-900 was determined at the constant current density of 10 mA cm^−2^, as shown in Figure 3d. It can be seen that there was no clear delay to the applied potential, proving that the as-synthesized HEO-900 sample had prominent operation durability. The high durability is attributed to the high-entropy stabilization effect of the (Co, Fe, Mn, Ni, Cr)_3_O_4_ catalysts. The above-mentioned results confirm that the as-prepared HEO-900 sample contains excellent OER activity and operating durability.

**Figure 4:**
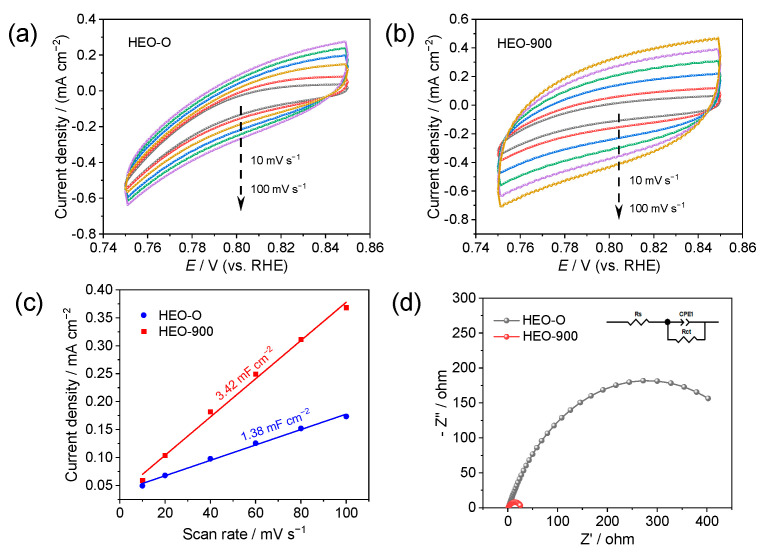
CV curves for (**a**) HEO-O and (**b**) HEO-900 at the scanning rates from 10 mV s^−1^ to 100 mV s^−1^. (**c**) Cdl values at various scanning rates. (**d**) Nyquist plots of HEO-O and HEO-900.

The CV curves were also determined to calculate the double-layer capacitance (Cdl) values of the HEO-O and HEO-900 samples, which were applied to investigate the electrochemical active surface area (ECSA) [37,38]. The CV curves were detected in the potential range of 0.75~0.85 V at the scanning rates from 10 mV s^−1^ to 100 mV s^−1^, as shown in Figure 4a,b. The Cdl values were calculated based on the CV curves, as shown in Figure 4c. It is noteworthy that the HEO-900 electrocatalyst contains a higher Cdl value of 3.42 mF cm^−2^ than that of HEO-O (1.38 mF cm^−2^). This result proves that the as-prepared sample of HEO-900 possesses a higher ECSA value, revealing the promoted exposure of OER active sites. Moreover, impedance is also a significant parameter for the electrocatalysts. As depicted in Figure 4d, the HEO-900 catalyst displayed a lower interface transfer impedance of 26.31 Ω than that of HEO-O (548.4 Ω), revealing the enhanced electron transfer. The low impedance is profitable for accelerating the OER process.

### 3.3. Catalytic Mechanism Analysis

**Figure 5:**
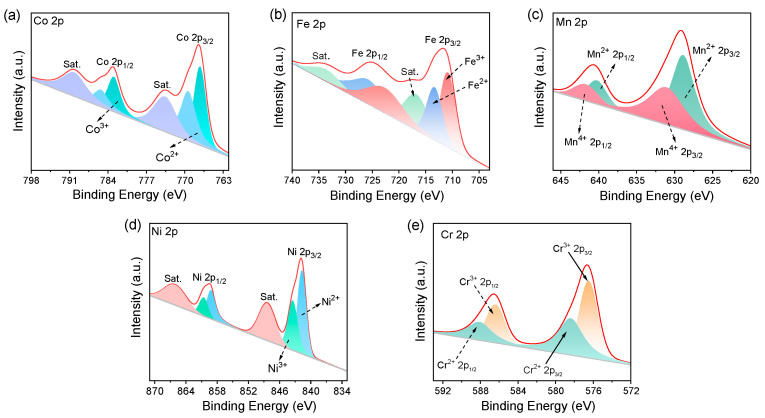
XPS spectra of HEO-900 after OER determination. (**a**) Co 2p spectrum. (**b**) Fe 2p spectrum. (**c**) Mn 2p spectrum. (**d**) Ni 2p spectrum. (**e**) Cr 2p spectrum.

In order to deeply exploit the catalytic mechanism, the surface element compositions and valence states of the HEO-900 catalyst after the OER process were determined by the XPS technique. The XPS full spectra indicate the coexistence of Ni, Co, Fe, Mn, Cr, and O elements in the HEO-900 catalyst (Figure S1). The high-resolution spectra of Co 2p, Fe 2p, Mn 2p, Ni 2p, and Cr 2p revealed that the bivalent and trivalent metal ions are presented for Co, Fe, Ni, and Cr elements, while the bivalent and quadrivalent metal ions were obtained for the Mn element, as depicted in Figure 5a–e. The high-valence metal species of Ni^3+^, Co^3+^, Fe^3+^, Mn^4+^ and Cr^3+^ were generated on the surface of the HEO-900 electrocatalyst after the OER. Generally, the high-valence metal species favor the adsorption of OER intermediates and are regarded as highly active phases for the OER process [39,40]. Here, the abundant metal sites of Co^3+^, Fe^3+^, Mn^4+^, Ni^3+^, and Cr^3+^ coexist on the HEO-900 catalyst surface, and their synergetic effect is conducive to enhancing the activity of OER. Thus, the spinel-type high-entropy oxides of (Co, Fe, Mn, Ni, Cr)_3_O_4_ achieve excellent OER performances.

## 4. Conclusions

In this work, we successfully synthesized the spinel-type high-entropy oxides of (Co, Fe, Mn, Ni, and Cr)_3_O_4_ through the high-temperature calcination approach. The effect of calcination temperatures on structures and properties was investigated systematically. It is noteworthy that the optimized catalyst of HEO-900 contains the hybrid structure of regular polyhedrons and irregular nanoparticles, which is beneficial for the exposure of electrochemically active sites. Owing to the synergistic effect of the multi-metal sites, the as-prepared HEO-900 catalyst indicates excellent OER activity and needs the overpotential of 366 mV to reach the current density of 10 mA cm^−2^. In addition, the HEO-900 catalyst exhibits the prominent durability of running for 24 h at the current density of 10 mA cm^−2^ with clear delay. Therefore, this work supplies a promising route for preparing high-performance multi-metal OER electrocatalysts for application in water electrocatalysis.

## Data Availability

The data presented in this study are available upon request from the corresponding author.

## Supplementary Materials

The following supporting information can be downloaded at: https://www.mdpi.com/article/10.3390/ma17143415/s1, Figure S1: XPS full spectrum of HEO-900 after the OER determination; Table S1: Comparison of OER performances of HEO-900 with the recently reported catalysts.
