# Supplementary material for: Fabricating Spinel-Type High-Entropy Oxides of (Co, Fe, Mn, Ni, Cr)3O4 for Efficient Oxygen Evolution Reaction
Source: Materials (Basel). 2024 Jul 10;17(14):3415. doi: 10.3390/ma17143415 (PMC11277610; doi:10.3390/ma17143415)
Supplement: Supplementary file 1 [file materials-17-03415-s001.zip › materials-3047661-supplementary.pdf]

Supporting information

Fabricating Spinel-Type High-Entropy Oxides of (Co, Fe, Mn, Ni, Cr)$_3$O$_4$ for Efficient Oxygen Evolution Reaction

Xiaofei Hao [1,*], Ran Wang [2], Xiumin Tan [1], Xiufeng Zhang [1], Xupo Liu [2,*], Zhaoyang Wu [1] and Dongli Yuan [1]

[1] Zhengzhou Institute of Multipurpose Utilization of Mineral Resources, Chinese Academy of Geological Sciences, Zhengzhou 450006, China; zzs_tan@163.com (X.T.); zh200318@126.com (X.Z.); wzy500@sina.com (Z.W.); yuandongli99@163.com (D.Y.)

[2] School of Materials Science and Engineering, Henan Normal University, Xinxiang 453007, China; 2125283034@stu.htu.edu.cn

* Correspondence: haopaper@163.com (X.H.); liuxupo@htu.edu.cn (X.L.); Tel.: +86-19837166511 (X.H.); +86-15907164432 (X.L.)

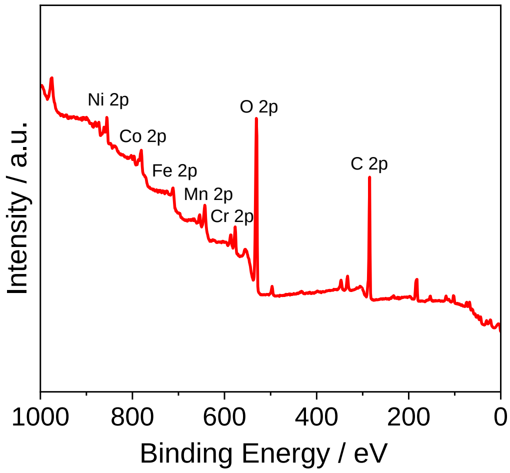

Figure S1. XPS full spectrum of HEO-900 after the OER determination.

Table S1. Comparison of OER performances of HEO-900 with the recently reported catalysts.

Electrocatalysts	Overpotential at 10 mA cm^{-2} (mV)	Tafel slope (mV dec^{-1})	References
HEO-900	366	76.35	This work
$Ni_{0.75}Cu_{0.25}Co_2O_4$	509	119	[1]
$CrCo_2O_4$	342	68.1	[2]
Ni-Co_3O_4	380	63.19	[3]
$FeMn_2O_4$	350	100.7	[4]
$CoCeZnO_x$	344	98	[5]
Co_3O_4-B-2	378	58.18	[6]
$NiFe_2O_4$	410	80	[7]
Mo-Co-Ni_3S_2 /NF	332	104	[8]
gC_3N_4/NiCoP/NF	370	64	[9]
Co/Mo-rGO	420	169	[10]
$LaNiO_3$-NiO	383	80.73	[11]
CoFeB	390	66.35	[12]

References

1. Park, H.; Park, B.H.; Choi, J.; Kim, S.; Kim, T.; Youn, Y.-S.; Son, N.; Kim, J.H.; Kang, M. Enhanced electrochemical properties and OER performances by Cu substitution in $NiCo_2O_4$ spinel structure. *Nanomaterials* **2020**, *10*, 1727, doi:https://doi.org/10.3390/nano10091727.
2. Lee, S.; Kim, S.; Jang, Y.; Park, J.; Byeon, J.; Lee, J. Oxophilicity induced surface hydroxylation to promote oxygen evolution in selectively substituted spinel-type cobalt oxides. *J. Phys. Chem. C* **2023**, *127*, 15062-15068, doi:https://doi.org/10.1021/acs.jpcc.3c02867.
3. Vazhayil, A.; Ashok C, S.; Thomas, N. Probing the electrocatalytic activity of hierarchically mesoporous M-Co_3O_4 (M = Ni, Zn, and Mn) with branched pattern for oxygen evolution reaction. *J. Electroanal. Chem.* **2023**, *934*, 117298, doi:https://doi.org/10.1016/j.jelechem.2023.117298.
4. Qi, C.; Liu, Q.; Dong, Y.; Zhang, G.; Jiang, X.; Gao, D. Quenching-induced surface reconstruction of $FeMn_2O_4$ for promoted oxygen evolution reaction. *J. Alloys Compd.* **2023**, *967*, 171754, doi:https://doi.org/10.1016/j.jallcom.2023.171754.

5. Huang, X.; Wang, X.; Liu, Y.; Hou, Y.; Li, C.; Cai, M.; Gu, H.; Cao, X. General synthesis of $CoCeMO_x$ trimetallic oxides via a cation exchange reaction for the oxygen evolution reaction. *Dalton Trans.* **2023**, *52*, 5312-5320, doi:https://doi.org/10.1039/D3DT00318C.
6. Guo, S.; Wang, X.; Zhou, X.; Li, H.; Ding, X. Tuning oxygen vacancies in Co_3O_4 nanorods through solvent reduction method for enhanced oxygen evolution activity. *Energy Fuels* **2023**, *37*, 5421-5428, doi:https://doi.org/10.1021/acs.energyfuels.3c00034.
7. Sultan, F.; González Sepúlveda, G.E.; Medina, D.I.; Videa, M.; Sánchez-Domínguez, M.; Cholula-Díaz, J.L. Synthesis of MFe_2O_4 (M=Ni, Co) nanoparticles by a bicontinuous microemulsion method for the oxygen evolution reaction. *ChemNanoMat* **2024**, *10*, e202300541, doi:https://doi.org/10.1002/cnma.202300541.
8. Shoaib, M.; Qiao, F.; Xu, X.; Zhou, T.; Liu, Y. Influence of Mo concentration on the structural and electrochemical properties of double-doped Mo-Co-Ni_3S_2/NF composites. *CrystEngComm* **2024**, *26*, 1884-1891, doi:https://doi.org/10.1039/D3CE01246H.
9. Kayış, Z.; Akyüz, D. A high-performance electrocatalyst via graphitic carbon nitride nanosheet-decorated bimetallic phosphide for alkaline water electrolysis. *Phys. Chem. Chem. Phys.* **2024**, *26*, 14908-14918, doi:https://doi.org/10.1039/D4CP00020J.
10. Zhao, L.; Liu, S.; Wei, L.; He, H.; Jiang, B.; Zhan, Z.; Wang, J.; Li, X.; Gou, W. One-pot hydrothermal synthesis of bifunctional Co/Mo-rGO efficient electrocatalyst for HER/OER in water splitting. *Catal. Lett.* **2024**, doi:https://doi.org/10.1007/s10562-024-04723-w.
11. Li, W.; Xu, H.; Pei, Y.; Hu, L.; Yang, Z. Investigation into the performance of tremella-like $LaNiO_3$-NiO composite as an electrocatalyst for oxygen evolution reaction. *Ionics* **2024**, doi:https://doi.org/10.1007/s11581-024-05614-1.
12. Wang, L.; Li, J.; Li, Y.; Dong, X.; Shan, H.; Chen, S.; Sun, P.; Zhang, F.; Li, W.; Chu, X.; et al. Cobalt-based transition metal boride electrocatalysts for alkaline water oxidation reactions. *Ionics* **2024**, *30*, 943-950, doi:https://doi.org/10.1007/s11581-023-05302-6.
